# Ising Paradigm in Isobaric Ensembles

**DOI:** 10.3390/e26060438

**Published:** 2024-05-22

**Authors:** Claudio A. Cerdeiriña, Jacobo Troncoso

**Affiliations:** Instituto de Física e Ciencias Aeroespaciais da Universidade de Vigo and Unidad MSMN Asociada al CSIC por el IQF Blas Cabrera, 32004 Ourense, Spain; jacobotc@uvigo.es

**Keywords:** Ising-like models, compressible cells, local entropic effects, *NpT* and *μpT* ensembles, asymmetric fluid criticality, water’s unusual thermodynamics, freezing

## Abstract

We review recent work on Ising-like models with “compressible cells” of fluctuating volume that, as such, are naturally treated in NpT and μpT ensembles. Besides volumetric phenomena, local entropic effects crucially underlie the models. We focus on “compressible cell gases” (CCG), namely, lattice gases with fluctuating cell volumes, and “compressible cell liquids” (CCL) with singly occupied cells and fluctuating cell volumes. CCGs contemplate singular diameters and “Yang–Yang features” predicted by the “complete scaling” formulation of asymmetric fluid criticality, with a specific version incorporating “ice-like” hydrogen bonding further describing the “singularity-free scenario” for the low-temperature unusual thermodynamics of supercooled water. In turn, suitable CCL variants constitute adequate prototypes of water-like liquid–liquid criticality and the freezing transition of a system of hard spheres. On incorporating vacant cells to such two-state CCL variants, one obtains three-state, BEG-like models providing a satisfactory description of water’s “second-critical-point scenario” and the whole phase behavior of a simple substance like argon. Future challenges comprise water’s crystal–fluid phase behavior and metastable states.

## 1. Background and Scope

### 1.1. Ising Model and Standard Lattice Gas

Understanding the microscopic basis of the distinct phases of matter and the transitions between them has long been recognized a major topic in Statistical Physics. Associated with it is the extraordinarily complex mathematical problem posed by a macroscopic number of interacting constituents. This is the reason why the focus has been put on the simplest models, which, remarkably, have been found to contain the essential physics. One such model is certainly the Ising model of ferromagnetism [1], whose solutions in d=2 [2,3,4] and d=3 dimensions [5] have triggered the development of the modern theory of critical phenomena [6,7,8]. Another relevant prototype is the XY model [9], which has featured topological phase transitions in d=2 [10]. A third prominent example is the Sherrington–Kirkpatrick model of a spin glass [11], whose solution has revealed the laws of disorder [12]. The models cited are most representative members of respective classes of models: while the Ising class is the simplest one and the first to be worked out, it has remained useful with time [13]. The present articles summarizes recent work indicating that the Ising paradigm continues to thrive. In doing so, we start by briefly reviewing the original Ising model.

**Figure 1:**
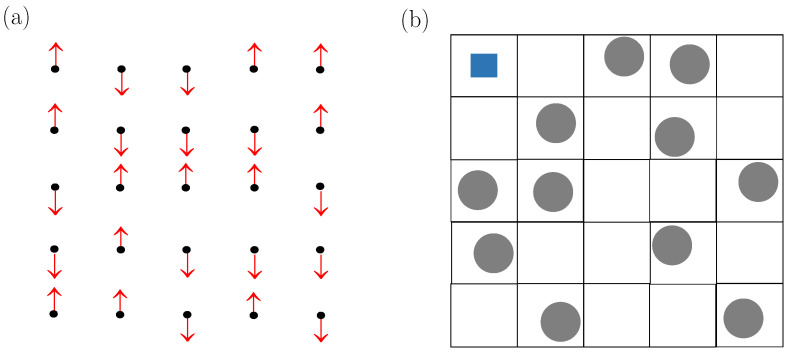
(**a**) Ising model in a square lattice. (**b**) Standard lattice gas of hard-core spherical particles in a square lattice. The blue-shaded area in the upper-left cell is the free volume ${\dot{v}}_{0}$ a particle explores according to the criterion explained in the text. More details about both models including the coupling between nearest-neighbor pairs are specified in Section 1.1.

Figure 1a illustrates that at each site of a *d*-dimensional regular lattice of coordination number *c*, there is a spin that can point either upwards (↑) or downwards (↓), with the energy of two neighboring spins decreasing by J>0 when they both are in the same individual state. This is known to result in a phase transition akin to the ferromagnetic–paramagnetic transition of an uniaxial ferromagnet. For a fixed number of sites N, only the system’s energy *E* fluctuates and so a canonical treatment is in order. Specifically, at temperature *T* and magnetic field *H*, the partition function is

(1) $$Z=\sum_{\left\{s_{i}\right\}}\exp\left(K\sum_{<ij>}s_{i}s_{j}+h\sum_{i=1}^{N}s_{i}\right),\;$$

where $s_{i}=-1,1$ applies when spin in site *i* is in a ↓,↑ state, <ij> refers to spins in nearest-neighbor sites, and

(2) $$\bar{f}\equiv -\frac{F}{Nk_{B}T}=\frac{\ln Z}{N},\;\;\;\;\;\;\;\;\;\;\;\;\;\;\;\;\;\;\;\;\;\;\;\;K\equiv \frac{J}{k_{B}T},\;\;\;\;\;\;\;\;\;\;\;\;\;\;\;\;\;\;\;\;\;\;\;\;h\equiv \frac{H}{k_{B}T},\;$$

with $k_{B}$ the Boltzmann constant and *F* the free energy.

The Ising model gained relevance in light of the pioneering work by Lee and Yang [14] proving its mathematical equivalence to the standard lattice gas (SLG) of condensation earlier introduced by Cernushi and Eyring [15]. This indicated the “Ising machinery” to underlie the phase behavior of systems of a distinct physical nature such as ferromagnets and fluids. This major idea of equivalence and universality lies behind the recent progress to be described in this article, to which the SLG is, besides, pivotal. It is then pertinent to review the SLG and its equivalence with the Ising model.

Figure 1b illustrates that, in the SLG, one considers that lattice sites are either vacant or occupied by one particle that interacts with particles in nearest-neighbor sites via a discrete energy $-\varepsilon_{0}<0$. This may be interpreted more realistically as a continuum model in which the *d*-dimensional space is divided into “cells” of volume $v_{0}$, each of which is associated with a site of the underlying lattice. Each particle explores a free volume ${\dot{v}}_{0}$ in its cell. A most elementary assumption is to consider that particles are allowed to move as if the cell boundaries act as impenetrable walls, which gives ${\dot{v}}_{0}\leq v_{0}$ with the equality holding for the marginal case of point particles. Note that walls are imaginary (rather than real) physical objects merely serving to provide a criterion to manage free volumes.

Clearly, for a given number of cells N, both the model’s *E* and number of particles *N* are allowed to fluctuate, with the volume $V=Nv_{0}$ fixed. A grand canonical (or μVT) treatment—with μ the chemical potential—then emerges naturally. The corresponding partition function is

(3) $$\Xi =\sum_{\left\{n_{i}\right\}}\exp\left(\bar{\beta }\varepsilon_{0}\sum_{<ij>}n_{i}n_{j}+\bar{\mu }\sum_{i=1}^{N}n_{i}\right),\;$$

where $n_{i}=0,1$ for vacant and occupied cells, $\bar{\beta }\equiv 1/k_{B}T$, and $\bar{\mu }\equiv \mu /k_{B}T-\ln\left(\Lambda^{3}/{\dot{v}}_{0}\right)$ with $\Lambda =\hbar \sqrt{2\pi /mk_{B}T}$ the thermal de Broglie wavelength for a particle with mass *m*. The groundbreaking observation by Lee and Yang was to show that (1) and (3) are equivalent with

(4) $$\bar{f}=\bar{p}v_{0}-\frac{c}{8}\bar{\beta }\varepsilon_{0}-\frac{1}{2}\bar{\mu },\;\;\;\;\;\;\;\;\;\;\;\;\;\;\;\;\;\;\;\;K=\frac{1}{4}\bar{\beta }\varepsilon_{0},\;\;\;\;\;\;\;\;\;\;\;\;\;\;\;\;\;\;\;\;h=\frac{1}{2}\bar{\mu }+\frac{c}{4}\bar{\beta }\varepsilon_{0},\;$$

where $\bar{p}\equiv p/k_{B}T$ with *p* the pressure. In purely mathematical terms, (4) is the exact analytic mapping of the SLG into the Ising model characterized by a one-to-one correspondence between ($\bar{f},K,h$) and ($\bar{p},\bar{\beta },\bar{\mu }$) that renders it an “isomorphism”. This result is useful because the solutions of the Ising model readily provide the ones of the SLG.

### 1.2. Decorated and BEG-like Models

**Figure 2:**
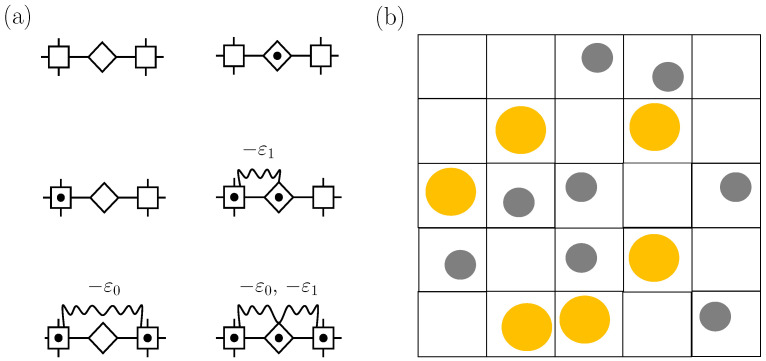
(**a**) Cell states and coupling of a decorated lattice gas with vertex cells (squares) and bond cells (diamonds). (**b**) BEG-like model with vacant cells and cells occupied by particles of two distinct species.

A valuable extension of the SLG entails a mathematical device known as the decoration transformation [5]. Figure 2a illustrates a decorated lattice gas that as such considers, in addition to vertex cells, cells in the bonds joining lattice sites. The model’s solutions are readily found from the ones for the SLG by merely evaluating the so-called decorating factors, which result from the summations over states for a bond cell. Note that the decorating system can be (as in Figure 2a) a cell that can be empty or occupied by one particle that interacts with particles in vertex cells, but one may generally consider an arbitrary statistical mechanical system [16]. This renders decorated lattice gases a flexible class of models. They have played a prominent role in fluid criticality [17].

The two-state nature of the SLG makes it suitable for describing a single phase transition. Models contemplating three states improve upon this insofar as they allow us to describe more than one phase transition and, hence, to account for a richer phenomenology. Figure 2b illustrates an early application for a two-component solution using vacant cells and cells containing particles of two distinct chemical species, the resulting model allowing us to account for gas–gas, gas–liquid, and liquid–liquid transitions [18]. Another relevant member of the class is a three-component model displaying intriguing phase behavior that includes tricriticality [19,20]. Progress on three-state models originated in part in work by Blume, Emery, and Griffiths [21], who introduced a variant jointly describing superfluid ordering and demixing in ${\mathrm{He}}^{3}$-${\mathrm{He}}^{4}$ solutions. As often carried out, we shall henceforth refer to these three-state variants as BEG-like (or simply BEG) models.

### 1.3. Compressible Cell Models

Common to the wide variety of models so far described, cells have a fixed volume $v_{0}$ that is the same for all cells. Progress accounted for in this review deals with the expediency of relaxing this constraint by allowing the individual cell volumes to fluctuate. This was pioneered by Stanley, Fisher, and coworkers for lattice gases [22,23,24,25], so that the resulting compressible cell gas (CCG) models incorporate local volumetric effects to the locally fluctuating number of particles and energy already contemplated. Inherent to fluctuating cell volumes are fluctuating free volumes carrying local changes in entropy *S*. On the other hand, local entropic effects associated with orientational degrees of freedom of molecules may also be considered [22]. In summary, there is the possibility to generate Ising-like models in which local fluctuations in energy, entropy, volume, and number of particles are coupled in a rich variety of ways.

Fluctuating cell volumes have opened the way to devise alternative models with all cells occupied [26,27], which will be henceforth referred to as compressible cell liquids (CCL). Of course, they may also suitably couple local energetic, entropic, and volumetric effects in distinct ways. A further crucial step is to build BEG-like models from them by simply adding vacant cells [27,28].

In what follows, we shall describe a variety of CCG, CCL, and BEG models addressing the topics of asymmetric fluid criticality, water’s unusual thermodynamics, and freezing transition of a simple substance, to which Section 2, Section 3 and Section 4 are, respectively, devoted. Each Section starts with the statement of the problem to then focus the attention on the specific nature of the corresponding models and the main results they lead to. Future challenges are introduced in Section 5.

Note that the statistical–mechanical analysis of CCL models is naturally made in the isothermal–isobaric (or NpT) ensemble, whereas CCG and BEG-like models demand a great grand canonical (or μpT) treatment. While the NpT ensemble has been widely used in simulations, it has left little effort for analytical work beyond the most basic textbook topic of ideal-gas thermodynamics and certain specific models of elasticity [29]. Even rarer is the use of the μpT ensemble, whose unconventional nature poses extra difficulties [27,30]. The present review summarizes most (if not all) work involving Ising-like models in these two isobaric ensembles.

## 2. Asymmetric Fluid Criticality

### 2.1. Complete Scaling with Singular Diameters and Yang–Yang Features

**Figure 3:**
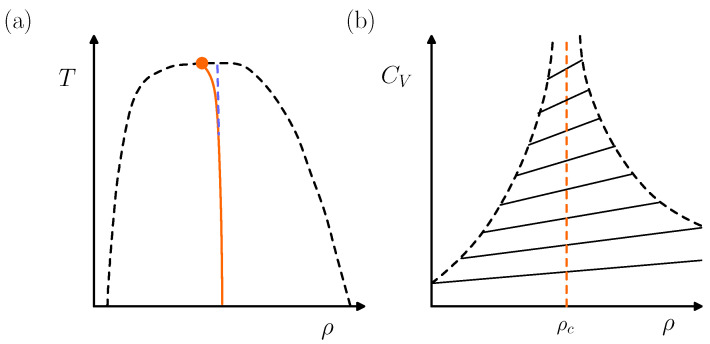
(**a**) Asymmetric gas–liquid coexistence curve of a fluid in the density–temperature ρ-*T* plane (dashed, black) ending at a critical point (circle, orange) with coordinates ($T_{c}$, $\rho_{c}$). The diameter (solid, orange) is the locus of the mid-points of the phase boundary and it curves in the immediate neighborhood of the critical point. According to complete scaling, the asymptotic curvature of the diameter is dominated by a $|T-T_{c}{|}^{2\beta }$ singularity. (**b**) Yang–Yang plot corresponding to the coexistence curve in (**a**). The isochoric heat capacity $C_{V}$ varies linearly with ρ at each $T<T_{c}$ (solid, black) within the two-phase envelope delimitated by the black dashed line. According to complete scaling, both the slope and the intercept diverge at criticality like $|T-T_{c}{|}^{-\alpha }$.

Early this century, experiment and simulation demanded the traditionally accepted scaling formulation of the thermodynamic behavior near the gas–liquid critical point to be revised [31,32]. The resulting theory, termed “complete scaling”, introduces additional terms in the expansions for each thermodynamic property [33]. A major effect is that the diameter of the coexistence curve in the density–temperature ρ-*T* plane varies asymptotically close to the critical temperature $T_{c}$ like $|T-T_{c}{|}^{2\beta }$, with β≃0.326. This implies an asymmetric coexistence curve close to criticality violating the classical Law of the Rectilinear Diameter: see Figure 3a for an schematic illustration. Associated with such an “asymmetric criticality” is a dilemma posed in 1964 by Yang and Yang [34] regarding the critical behavior of the isochoric heat capacity $C_{V}$ (see Figure 3b). The question arises whether there are Ising-like models exhibiting such “Yang–Yang and related features”.

The need for a prototype of gas–liquid criticality with known “exact” (i.e., non-mean-field) solutions initially puts the focus on the SLG. Nevertheless, the model is itself intrinsically irrelevant since it exhibits a trivial symmetry reminiscent to that for the spontaneous magnetization curve of the underlying Ising model upon magnetic field reversal H→−H. Moreover, while the decorated lattice gas of Figure 2a displays an asymmetric coexistence curve [35], it introduces a weaker $|T-T_{c}{|}^{1-\alpha }$ singularity in the diameter, with α≃0.109 and so 2β<1−α. This situation likewise occurs for the Widom–Rowlinson model of penetrable spheres [36] and Mermin’s “bar” model [37]. In this context, how can the SLG and usual variants be repaired for a $|T-T_{c}{|}^{2\beta }$ singularity in the diameter to come up? In practice, the task is to obtain the Ising ordering field *h* to depend on *p*. We shall explain below that CCG models meet this requirement. Moreover, we shall state the conditions by which CCGs mix all three physical fields *p*, *T*, and μ into *h* and *K*, thereby obeying complete scaling in all its aspects.

### 2.2. Most Basic Compressible Cell Gases

A first expediency is to simply allow the individual cell volumes of the SLG to fluctuate *freely* by, say, supposing that any cell can take *n* discrete volumes $0<v_{k}\leq v_{0}$ and a set of associated free volumes $0<{\dot{v}}_{k}<v_{k}$ (k=1,…,n) [24,25]. Clearly, *E*, *V*, and *N* fluctuate simultaneously for such a most basic ${\mathrm{CCG}}_{0}$ model, the μpT partition function being

(5) $$\Theta =S_{00}^{N}\sum_{\left\{n_{i}\right\}}\exp\;\left\{\bar{\beta }\varepsilon_{0}\sum_{<ij>}n_{i}n_{j}+{\left[\bar{\mu }+\ln\left(\frac{S_{01}}{v_{0}S_{00}}\right)\right]}^{\sum_{i=1}^{N}n_{i}}\right\},\;$$

with

(6) $$S_{00}\left(\bar{p}\right)=\frac{1}{n}\sum_{k=1}^{n}e^{-\bar{p}v_{k}},\;\;\;\;\;\;\;\;\;\;\;\;\;\;\;\;S_{01}\left(\bar{p}\right)=\frac{1}{n}\sum_{k=1}^{n}{\dot{v}}_{k}e^{-\bar{p}v_{k}}.\;$$

The following analytic mapping into the Ising model is found:

(7) $$\bar{f}=-\frac{1}{2}\ln\left[\frac{S_{01}S_{00}}{v_{0}}\right]-\frac{c}{8}\bar{\beta }\varepsilon_{0}-\frac{1}{2}\bar{\mu },\;$$

(8) $$K=\frac{1}{4}\bar{\beta }\varepsilon_{0},\;\;\;\;\;\;\;\;\;\;\;\;\;\;\;\;\;\;\;\;h=\frac{1}{2}\bar{\mu }+\frac{c}{4}\bar{\beta }\varepsilon_{0}+\frac{1}{2}\ln[\frac{S_{01}}{v_{0}S_{00}}],\;$$

which, via the explicit dependence of $S_{01}$ and $S_{00}$ on *p*, mixes the pressure into *h* as required for Yang–Yang features to show up.

One has the freedom to choose the cell volumes and free volumes in distinct ways. A most relevant result in this connection is that Yang–Yang features are absent in ${\mathrm{CCG}}_{0}$ models when ${\dot{v}}_{k}$ are the same for all *k*. This clearly renders the fluctuating free volumes explored by particles a source of Yang–Yang features. Moreover, the diameter curves towards higher densities as $T\to T_{c}$ when larger cell volumes are accompanied by larger free volumes, whereas it curves towards lower densities when free volumes are anticorrelated with cell volumes. The former case is realized by particles with a fixed core volume, the latter by compressible particles. Additional features such as changes in cell shape are also relevant.

While ${\mathrm{CCG}}_{0}$ models mix *p*, *T* and μ into *h*, they lack the mixing of *p* and μ into *K* demanded by complete scaling [see (8)]. Decorated ${\mathrm{CCG}}_{0}$s, namely, models such as the one in Figure 2a but with fluctuating cell volumes, fill the gap. The mapping of (7) and (8) is then supplemented by the decorating factors when vertex cells are both occupied $\Psi_{++}$, both vacant $\Psi_{--}$, and only one occupied $\Psi_{+-}$ to obtain

(9) $$\bar{f}=-\frac{1}{2}\ln\left[\frac{S_{01}S_{00}}{v_{0}}\right]-\frac{c}{8}\bar{\beta }\varepsilon_{0}-\frac{1}{2}\bar{\mu }-\frac{c}{8}\ln\left(\Psi_{++}\Psi_{--}\Psi_{+-}\right),\;$$

(10) $$K=\frac{1}{4}\bar{\beta }\varepsilon_{0}+\frac{1}{4}\ln\left(\frac{\Psi_{++}\Psi_{--}}{\Psi_{+-}^{2}}\right),\;\;\;\;\;\;\;\;\;\;\;\;\;\;\;\;\;\;\;\;h=\frac{1}{2}\bar{\mu }+\frac{c}{4}\left[\bar{\beta }\varepsilon_{0}+\ln\left(\frac{\Psi_{++}}{\Psi_{--}}\right)\right]+\frac{1}{2}\ln\left[\frac{S_{01}}{v_{0}S_{00}}\right].\;$$

Now, it is known since long ago [35] that bond cells with a fluctuating number of particles make $\Psi_{++}$, $\Psi_{--}$, $\Psi_{+-}$ and, hence, *K* to depend on μ. Further allowing the volume of decorating cells to fluctuate introduces *p* into $\Psi_{++}$, $\Psi_{+-}$, $\Psi_{--}$ and, hence, into *K*. Therefore, a decorated model with fluctuating volume and number of particles for both vertex and bond cells constitutes a sufficiently general model exhibiting all effects contemplated by complete scaling.

## 3. Water’s Unusual Thermodynamics

### 3.1. Singularity-Free Scenario Versus Second Critical Point

**Figure 4:**
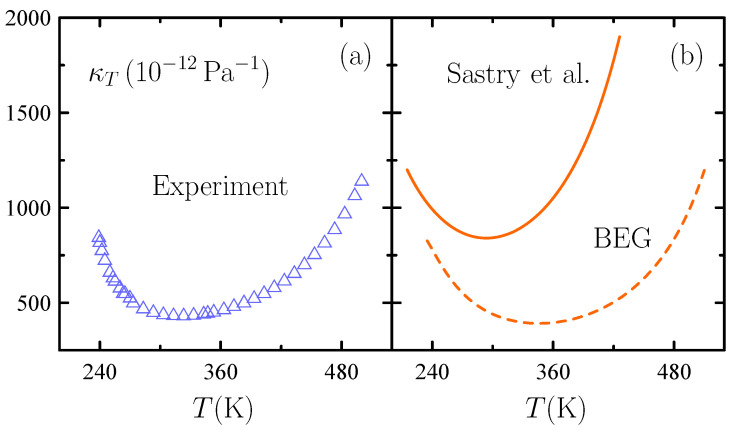
Isothermal compressibility $\kappa_{T}$ of water as a function of temperature *T* at atmospheric pressure. (**a**) Experimental data [38]. (**b**) Values from Sastry et al. [22] (solid, orange) and BEG-like (dashed, orange) models described in Section 3.2 and Section 3.3 with parameter settings in the original sources.

Groundbreaking experiments published in the 1970s and 1980s [38,39,40] evidenced a dramatic increase in the magnitude of thermodynamic response functions such as the isothermal compressibility $\kappa_{T}$, the isobaric heat capacity $C_{p}$, and the isobaric thermal expansivity $\alpha_{p}$ of supercooled water as *T* is lowered at atmospheric *p* below the freezing point: see Figure 4. Of the various competing physical pictures accommodating such unusual thermodynamic behavior [41,42], two of them largely survive nowadays: the so-called “second-critical-point” [43] and “singularity-free” [22,44] scenarios. The former hypothesizes that the enhancement of response functions is associated with the existence of a second, liquid–liquid critical point located in the region where water can exist as a deeply supercooled liquid. The latter merely points out that a second critical point is not necessarily demanded.

Recent state-of-the-art spectroscopic experiments point towards the existence of a second critical point [45]. Moreover, the second critical point has been proved via molecular simulation by Debenedetti, Sciortino, Poole, and coworkers for the ST2 model of water [46] and the even more accurate TIP4P/2005 and TIP4P/Ice [47], while the class of models exhibiting such behavior is rapidly expanding. In any event, what is the Ising expression for the singularity free versus second-critical-point dichotomy? Short answer is that suitable CCG and CCL models yield the singularity-free and second-critical-point scenarios, respectively.

### 3.2. Compressible Cell Gas with “Ice-like” Hydrogen Bonding

**Figure 5:**
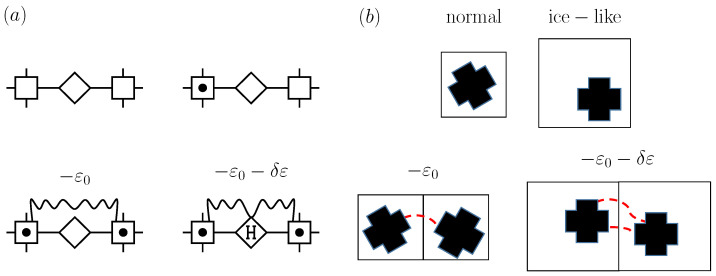
Cell states and pair couplings. (**a**) Sastry et al. decorated CCG described in Section 3.2. Note that the decorating system is, in contrast to the one in Figure 2a, a statistical–mechanical system implemented with the local energetic, entropic, and volumetric costs associated with ice-like hydrogen bonding in water. (**b**) Water-like CCL described in Section 3.3 with ice-like hydrogen bonding implemented through the standard Ising scheme.

Sastry et al. [22,23] pioneered a decorated model with vertex cells like in the SLG and bond cells mimicking the “ice-like structures” characteristic of liquid water at low temperatures. Specifically, as Figure 5a shows, bond cells serve to characterize the energetic, entropic, and volumetric features of ice-like hydrogen bonding: such bond cells account for the *q* distinct orientations for a single particle and further contemplate that only *q* of the $q^{2}$ possible orientations of two particles in nearest-neighbor cells lead to a hydrogen bond that is accompanied by an energy decrease and a volume increase in magnitudes δε and δv. This is a decorated CCG with vertex cells of fixed volume and bond cells of fluctuating volume. Note that, in contrast to ${\mathrm{CCG}}_{0}$ models, bond cell volume fluctuations are coupled to energy fluctuations. The concept is that the bond cell volume fluctuations set out relevant changes in intermolecular distances over which a pair potential acts.

The model may be worked out in the μpT ensemble since *E*, *V*, and *N* are fluctuating. The partition function is

(11) $$\Theta =\exp\left(-\bar{p}Nv_{0}\right)\sum_{\left\{n_{i}\right\}}\exp\{\left[\bar{\beta }\varepsilon_{0}+\ln\left(\frac{\Psi_{++}\Psi_{--}}{\Psi_{+-}^{2}}\right)\right]\sum_{<ij>}n_{i}n_{j}+c\ln(\frac{\Psi_{++}}{\Psi_{--}})\sum_{i=1}^{N}n_{i}\},\;$$

with

(12) $$\Psi_{--}=1,\;\;\;\;\;\;\;\;\;\;\;\;\;\;\;\;\;\;\;\;\Psi_{+-}=q,\;\;\;\;\;\;\;\;\;\;\;\;\;\;\;\;\;\;\;\;\Psi_{++}=q^{2}-q+qe^{\bar{\beta }\delta \varepsilon -\bar{p}\delta v}.\;$$

An exact analytic mapping into the Ising model comes out by simply inserting (12) into (9) and (10), with $S_{00}=e^{-\bar{p}v_{0}}$ and $S_{01}={\dot{v}}_{0}e^{-\bar{p}v_{0}}$ owing to the fixed-volume nature of vertex cells. [This is accompanied by Yang–Yang features because *p* enters into *h* thorugh $\Psi_{++}$.]

Since decoration is known to result in no additional phase transition [5], the gas–liquid transition of the underlying SLG is the only model’s transition. On the other hand, at low temperatures, significantly lower than the one of gas–liquid criticality, the Sastry et al. model predicts the magnitude of response functions to increase markedly as *T* is lowered just as observed experimentally for water: see Figure 4 for $\kappa_{T}$. Note that results of Figure 4 correspond to a mean-field calculation since no exact solutions for the underlying Ising model are available for the isobaric path of atmospheric pressure of interest (that is, h≠0). While a mean-field treatment is only exact in d=∞, there is a tacitly accepted consensus regarding it a reasonable approximation in d=3—from van der Waals or Curie–Weiss’ early work to the latest theory of simple glasses [48].

### 3.3. Compressible Cell Liquid with “Ice-like” Hydrogen Bonding and BEG Water-like Model

Ice-like structures can be alternatively implemented in another type of Ising-like model [26]. Thus, as Figure 5b shows, instead of occupied and vacant cells, one may consider a compressible cell liquid (CCL) with cells occupied by particles with no spherical symmetry and two distinct accessible volumes, $v_{0}$ and $v_{0}+\delta v$. Then, one further assumes that particles in bigger cells are in one of the *q* preferred orientations, while particles in smaller cells may display a full range of $q^{2}$ orientations. The cells with higher volume and lower entropy mimic the geometrical constraints of ice-like structures and are stabilized by a hydrogen-bonding energy −δε<0 when any two such “ice-like cells” are adjacent.

While fluctuating volumes serve in the Sastry et al. model to consider two distinct length scales over which an intermolecular potential acts, here the volume fluctuation concerns primary (instead of bond) cells of the Ising lattice. As such, the present model may be regarded a CCL model featuring the local energetic, entropic, and volumetric effects of water’s ice-like order with the aid of an Ising-like mechanism in which, statistically, higher volume and lower entropy per particle are accompanied by lower energy. This contrasts with the SLG, characterized by an Ising-like mechanism in which, statistically, a higher number of particles per volume are accompanied by lower energy. However, the “Ising machinery” is common to both models.

An NpT treatment is natural, with the partition function being

(13) $$\Delta ={\left(\frac{q^{2}{\dot{v}}_{0}}{\Lambda^{3}}\right)}^{N}\exp\left(-\frac{c}{2}\bar{\beta }\varepsilon_{0}-\bar{p}Nv_{0}\right)\sum_{\left\{n_{i}\right\}}\exp\left(\bar{\beta }\delta \varepsilon \sum_{<ij>}n_{i}n_{j}+\left(\ln\omega -\bar{p}\delta v\right)\sum_{i=1}^{N}n_{i}\right),\;$$

where N=N and ω=1/q quantify the entropy difference between ice-like and “normal” cells. This maps into (1) so that

(14) $$\bar{f}=\bar{p}\left(v_{0}+\frac{1}{2}\delta v\right)-\frac{1}{2}\ln\omega -\bar{\mu }-\frac{c}{2}\bar{\beta }\left(\varepsilon_{0}+\frac{1}{4}\delta \varepsilon \right),\;$$

(15) $$K=\frac{1}{4}\bar{\beta }\delta \varepsilon ,\;\;\;\;\;\;\;\;\;\;\;\;\;\;\;\;\;\;\;\;\;h=-\frac{1}{2}\bar{p}\delta v+\frac{1}{2}\ln\omega +\frac{c}{4}\bar{\beta }\delta \varepsilon .\;$$

**Figure 6:**
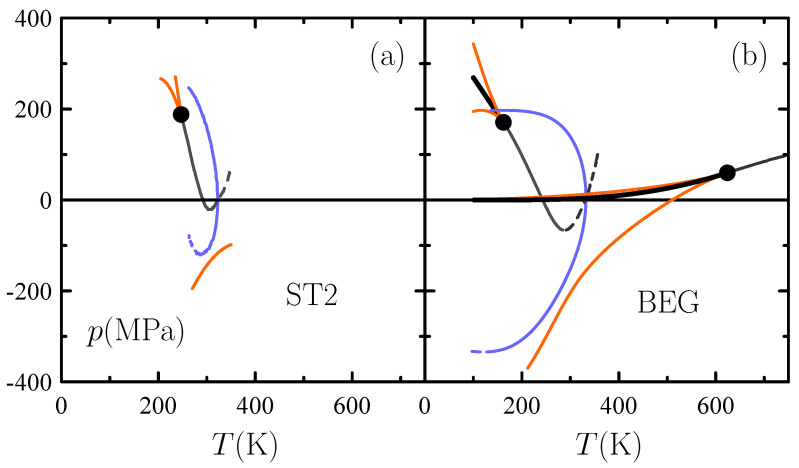
Phase diagram of models of fluid water in the pressure–temperature *p*-*T* plane. Plotted are the binodals (thick, black) and spinodals (solid, orange) associated with critical points (circles) at high *T* (gas–liquid) and low *T* (liquid–liquid), as well the locus of isobaric ρ(T) maxima (solid, blue) and minima (dashed, blue) and the locus of isobaric $\kappa_{T}\left(T\right)$ maxima (solid, grey) and minima (dashed, grey) (**a**) ST2 water [49]. (**b**) BEG water-like model described in Section 3.3.

The one-to-one correspondence between $\left(\bar{f},K,h\right)$ and $\left(\bar{p},\bar{\beta },\bar{\mu }\right)$ in (14) and (15) indicates that this CCL is isomorphic to the Ising model, implying that it leads to a phase transition terminating at a critical point. The transition may be thought of as involving two liquid phases: a low-density liquid phase rich in ice-like cells and a high-density liquid phase rich in normal cells. Since ice-like cells carry lower orientational entropy, *S* is lower for the low-density liquid and so the Clapeyron equation dictates a negative slope of the coexistence line in the *p*-*T* plane as it occurs for the ST2 model of water (see Figure 6).

Now, the mere expediency of adding vacant cells further incorporates the gas–liquid transition to our description through the standard SLG scheme. The resulting BEG-like model [28] demands a μpT treatment. On the other hand, it cannot be mapped into a model with known exact solutions, implying that a mean-field treatment is in order. Even within the uncertainties inherent to the approximate nature of a mean-field calculation for d=3, the model does account qualitatively for water’s second-critical-point scenario: see Figure 6. The idea of appealing to the BEG paradigm to account for water’s fluid-phase behavior was originally explored by Ciach et al. [50], who devised a version distinct from the one presented here.

Note that BEG-like and Sastry et al. models are essentially equivalent as far as the gas–liquid transition is concerned, while both employ compressible cells. The main difference between them lies on the way ice-like structures are implemented. Thus, the second-critical-point scenario demands a three-state variant combining SLG and CCL components, whereas the singularity-free scenario occurs in a (two-state) decorated CCG. This is the Ising expression of the singularity-free versus second-critical-point dichotomy.

**Figure 7:**
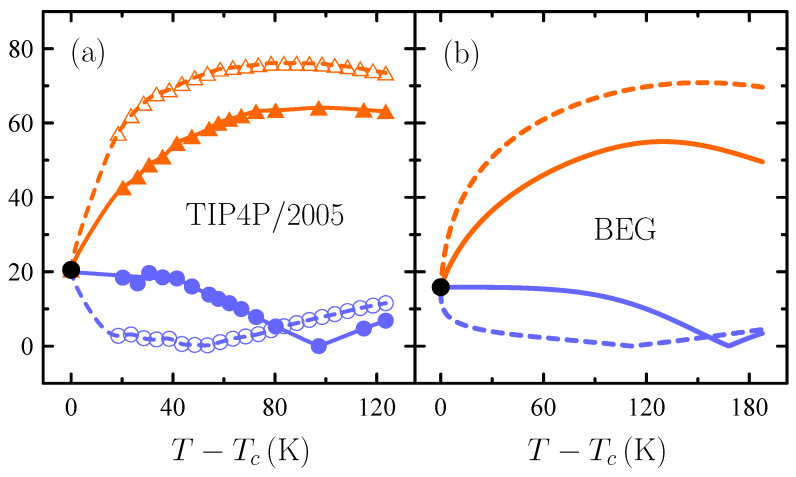
Response-function ratios $C_{p}/Tv\kappa_{T}$ (two upper curves, orange) and $|\alpha_{p}/\kappa_{T}|$ (two lower curves, blue) as a function of temperature *T* on approaching the liquid–liquid critical temperature $T_{c}$ (circles, black) from the one-phase region along the critical isochore (solid) and critical isobar (dashed) [51]. Results are in $\mathrm{bar}\;\;K^{-1}$ (**a**) TIP4P/2005 water (points are simulation data). (**b**) BEG water-like model.

Regardless of which scenario really corresponds to water, analyzing liquid–liquid criticality has an intrinsic interest as a theoretical possibility. In this connection, Figure 7 certifies that the BEG-like model provides a pattern of liquid–liquid critical behavior consistent with the one for TIP4P/2005 water. An experimental verification of this remains a great challenge, but before answers in any direction are obtained, Figure 7 may be a reference as to what should be expected.

## 4. Freezing

### 4.1. An Open Problem to Statistical Mechanics

While the transition between solid and fluid phases is well-known experimentally since long ago, its statistical–mechanical characterization remains an outstanding question to the theory of condensed matter [52]. The theory of topological order is able to describe melting in d=2 [53], while classical density functional theory has proven efficient for computing solid–fluid phase diagrams [54]. Nevertheless, no definite theory for d=3 currently exists.

Early work by Lennard-Jones and Devonshire [55,56] claimed cell models to describe freezing, but the idea was later abandoned and seemingly fell into oblivion [57]. A major breakthrough stemmed from 1957 pioneering molecular simulations [58,59] unexpectedly indicating the hard-sphere fluid to freeze into a face-centered cubic crystal. Such a piece of evidence was found and discussed at a time at which the Ising model and the SLG were becoming widely accepted. It was then natural to inquire which sort of Ising-like variant was needed to describe hard-sphere freezing. To answer it, much attention was placed on the class of “hard-core lattice particle” models [60,61,62,63]. Nevertheless, they have only led to a strictly first-order transition conforming to the nature of freezing (i.e., no critical point) when interactions beyond first neighbors are contemplated [64,65,66,67]. The question arises whether or not there is a reasonable Ising-like model accounting for freezing in a first-neighbor scheme, just as it occurs with the SLG for condensation. A suitable CCL model answers this question positively, and with further incorporation of attractive interactions, it leads to the whole phase behavior of a simple substance like argon.

### 4.2. Compressible Cell Liquid with Molecular Packing

**Figure 8:**
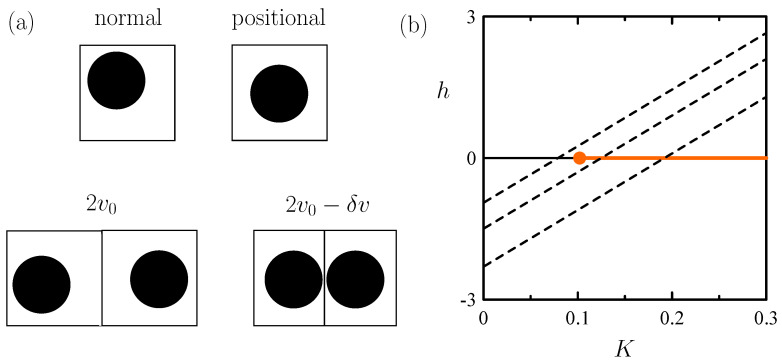
CCL model for molecular packing described in Section 4.2. (**a**) Cell states and coupling between nearest-neighbor pairs. (**b**) Ising (*K*, *h*) state points mapped by the model (dashed, black straight lines) corresponding to ω values decreasing from top to bottom. They cross the Ising transition line (thick, orange) when ω is small enough. Thus, beyond the borderline case in which the crossing point is at $K=K_{c}$, the transition is strictly first-order.

Let us consider a CCL with singly occupied cells as in Section 3.3, but distinguish two distinct situations for the free volumes that a hard sphere of diameter σ can explore in its cell [27]. As Figure 8a shows, we are led to think, as described in Section 1.1 for the SLG, about standard cells with free volume ${\dot{v}}_{0}$ constrained by σ and the cell boundaries. In addition, one contemplates a situation of “positional order” (and, hence, lower entropy) in which a particle explores a preferential, restricted free volume ${\dot{v}}_{1}<{\dot{v}}_{0}$ around the center of its cell. Our description is then completed by simply postulating that the total volume of an assembly of two nearest-neighbor cells is decreased by δv>0 when they both have the prescribed positional order (see Figure 8a). This CCL features molecular packing via an Ising-like mechanism in which, statistically, lower entropy per particle is accompanied by a lower volume. The concept is that a most efficient packing can only be achieved when particles occupy preferred positions in space.

The model is naturally treated in the NpT ensemble. The partition function is

(16) $$\Delta ={\left(\frac{{\dot{v}}_{0}}{\Lambda^{3}}\right)}^{N}\exp\left(-\bar{p}Nv_{0}\right)\sum_{\left\{n_{i}\right\}}\exp\left(\bar{p}\delta v\sum_{<ij>}n_{i}n_{j}+\ln\omega \sum_{i=1}^{N}n_{i}\right),\;$$

with N=N and $\omega ={\dot{v}}_{1}/{\dot{v}}_{0}$ serving to quantify the entropy difference between standard and positional cells. A comparison with (1) yields the following mapping:

(17) $$\bar{f}=\bar{p}\left(v_{0}-\frac{c}{8}\delta v\right)-\frac{1}{2}\ln\omega -\bar{\mu },\;\;\;\;\;\;\;\;\;\;\;\;\;\;\;\;\;\;\;\;\;K=\frac{1}{4}\bar{p}\delta v,\;\;\;\;\;\;\;\;\;\;\;\;\;\;\;\;\;\;\;\;\;h=\frac{1}{2}\ln\omega +\frac{c}{4}\bar{p}\delta v.\;$$

This is essentially different from all mappings described previously since both *K* and *h* just depend on a single variable, namely, $\bar{p}$. Thus, no one-to-one correspondence between ($\bar{f}$, *K*, *h*) and ($\bar{p}$, $\bar{\beta }$, $\bar{\mu }$) holds, and as Figure 8b shows, only a straight line in the (*K*, *h*) space is covered. In purely mathematical terms, this mapping is a monomorphism rather than an isomorphism. Furthermore, the conditions leading to a strictly first-order transition (i.e., no critical point) are illustrated by Figure 8b. Fulfillment of such conditions meets a distinguishing feature of freezing, as revealed by Landau’s theoretical statements and all known experimental evidence.

### 4.3. BEG-like Model and Van Der Waals Picture

**Figure 9:**
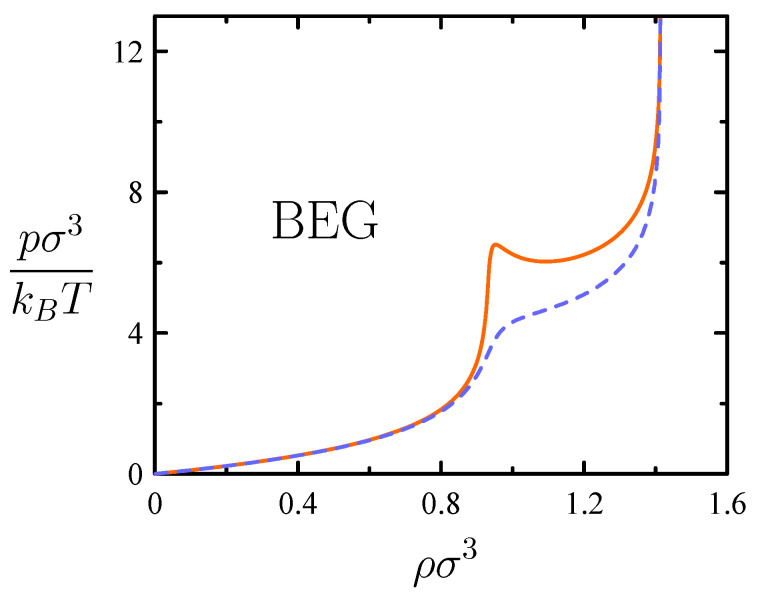
Behavior of BEG-like model for packing of hard spheres of diameter σ described in Section 4.2. A van der Waals loop appears for ω=0.1 (solid, orange), whereas it is absent for ω=0.2 (dashed, blue) [cf. discussion in the caption of Figure 8].

Adding vacant cells results in a BEG-like model allowing us to describe the range of states of lower density ρ towards the ideal-gas limit [27]. A concomitantly fluctuating *N* then naturally leads to the μpT ensemble. Moreover, as noted in Section 3.3, the BEG-like model demands a mean-field treatment. This yields the $p\sigma^{3}/k_{B}T$ versus $\rho \sigma^{3}$ curve of Figure 9 contemplating a van der Waals loop as the signature of a phase transition. The purely-repulsive nature of interactions considered renders reasonable to identify the model’s transition as the freezing transition of hard spheres proved by molecular simulation.

Following up on the model for the hard-sphere system, it is natural to inquire whether incorporation of attractive interactions in a way consistent with a normal pair potential like the one due to Lennard-Jones and Devonshire yields the phase behavior known experimentally for a simple fluid like argon. Such a desirable implementation may be readily accomplished by simply supposing that there is a background interaction energy for particles in nearest-neighbor cells $-\varepsilon_{0}<0$ that is supplemented by an additional energy −δε<0 when both cells have positional order. This mimics the minimum of a potential well.

**Figure 10:**
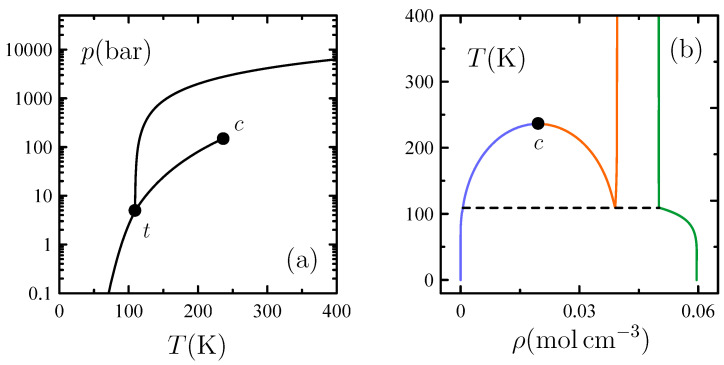
Phase diagram for the simple-substance model described in Section 4.3. (**a**) Pressure–temperature *p*-*T* plane. (**b**) Temperature–density *T*-ρ plane. Lines in the *p*-*T* plane determine the conditions of two-phase coexistence bounded by the triple point *t* and the gas–liquid critical point *c*. Lines in the *T*-ρ plane enclose regions of two-phase coexistence for crystal (solid, green), liquid (solid, orange), and gas (solid, blue), with horizontal dashed lines joining the states of three-phase coexistence associated with *t*.

Figure 10 shows that the resulting phase diagrams in the *p*-*T* and *T*-ρ planes meet all essential features observed experimentally. Perturbatively adding attractive interactions to a reference hard-sphere system therefore proves efficient for describing the whole phase behavior of a simple substance. This idea underlay the early work by van der Waals for the gas–liquid transition and was later extended to freezing by Longuet-Higgins and Widom [68], who, however, started from the corrected equation of state of hard spheres provided by molecular simulation. The BEG model with compressible cells and simple attractive interactions wholly develops such a contemporary van der Waals picture [69,70] by preliminary meeting the hard-sphere phenomenology from first principles of statistical mechanics.

## 5. Future Work

A natural extension of the work summarized above points toward water’s phase behavior. Of course, one may not realistically expect to cover the many (more than 15) crystalline ices. Nevertheless, on the basis of what we already have for the freezing of a simple liquid, a reasonable incorporation of some crystal phase to the current description of the fluid phases seems feasible.

Another line of inquiry is metastability. This topic can be approached from mean-field theory since it supports the existence of thermodynamic metastable states [48,71]. Of significant interest are the various metastable two-phase equilibria inherent to the existence of three phases [72]. Likewise appealing are spinodal curves, as the mean-field limit of the regions of thermodynamic space where each phase can exist. A primary objective is to find an overall picture consistent with evidence known from experimentation and simulation. In this connection, a major challenge is to explain the absence of a supercooled liquid spinodal. Has mean-field theory anything useful to say about this?

In our own preliminary assessment, adequate answers regarding the absence of a supercooled liquid spinodal may arise from the tools so far described in the present review article. This issue is certainly relevant for water, with its hypothesized metastable liquid–liquid transition in the supercooled region. A consistent mean-field implementation of such a transition as a liquid–liquid equilibrium metastable with respect to the crystal would certainly be an ultimate goal of the work summarized here on the Ising paradigm in isobaric ensembles as associated with the concept of compressible cells.

## Abbreviations

The following abbreviations are used in this manuscript:

SLG	Standard lattice gas
CCG	Compressible cell gas
CCL	Compressible cell liquid
BEG	Blume–Emery–Griffiths
